# Feasibility and safety of deep sedation with propofol and remifentanil in spontaneous breathing during endoscopic retrograde cholangiopancreatography: an observational prospective study

**DOI:** 10.1186/s12871-023-02218-6

**Published:** 2023-08-04

**Authors:** Pasquale De Vico, Daniele G. Biasucci, Lucia Aversano, Roberto Polidoro, Alessia Zingaro, Francesca Romana Millarelli, Giovanna Del Vecchio Blanco, Omero Alessandro Paoluzi, Edoardo Troncone, Giovanni Monteleone, Mario Dauri

**Affiliations:** 1 Department of Clinical Science and Translational Medicine, ‘Tor Vergata’ University of Rome, Rome, Italy; 2 Emergency Department, ‘Tor Vergata’ University Hospital of Rome, Rome, Italy; 3 Department of Systems Medicine, ‘Tor Vergata’ University of Rome, Rome, Italy

**Keywords:** Total intravenous anesthesia, Deep sedation, Endoscopic retrograde cholangiopancreatography, Nonoperating room anaesthesia

## Abstract

**Background:**

Endoscopic retrograde cholangiopancreatography (ERCP) is an interventional procedure that requires deep sedation or general anaesthesia. The purpose of this prospective observational study was to assess the feasibility and safety of deep sedation in ERCP to maintain spontaneous breathing.

**Methods:**

This is a single-centre observational prospective cohort study conducted in a tertiary referral university hospital. All consecutive patients who needed sedation or general anaesthesia for ERCP were included from January 2021 to June 2021. Deep sedation was achieved and maintained by continuous infusion of an association of propofol and remifentanil. The primary endpoint was to assess the prevalence of major anaesthesia-related complications, such as arrhythmias, hypotension, gas exchange dysfunction, and vomiting (safety endpoint). Secondary endpoints were: (a) to assess the prevalence of signs of an insufficient level of sedation, such as movement, cough, and hiccups (feasibility endpoint): (b) time needed to achieve the target level of sedation and for recovery from anaesthesia. In order to do so we collect the following parameters: peripheral oxygen saturation, fraction of inspired oxygen, noninvasive systemic blood pressure, heart rate, number of breaths per minute, neurological functions with the use of the bispectral index to determine depth of anaesthesia, and partially exhaustive CO_2_ end pressure to continuously assess the ventilatory status. The collected data were analysed by several tests: Shapiro-Wilk, Student’s t, Tuckey post-hoc, Wilcoxon rank-sum and Kruskall-Wallis ran. Statistical analysis was performed using Stata/BE 17.0 (StataCorp LLC).

**Results:**

114 patients were enroled. Eight patients were excluded because they did not meet the inclusion criteria. We found that all patients were hemodynamically stable: intraoperative mean systolic blood pressure was 139,23 mmHg, mean arterial pressure was on average 106,66 mmHg, mean heart rate was 74,471 bpm. The mean time to achieve the target level of sedation was 63 s, while the mean time for the awakening after having stopped drug infusion was 92 s.

**Conclusions:**

During ERCP, deep sedation and analgesia using the association of propofol and remifentanil and maintaining spontaneous breathing are safe and feasible, allowing for a safe and quick recovery from anaesthesia.

## Background

Endoscopic retrograde cholangiopancreatography (ERCP) is a complex endoscopic procedure performed to investigate conditions that affect the biliary tree and pancreas and for therapeutic interventions. The degree of complexity of ERCP can vary considerably, and increased complexity affects procedural times, technical success, and the appearance of adverse events [1–7].

To improve the success rate of the procedure, reduce the risk of adverse events, and maximise patient comfort, ERCP is generally performed under deep sedation (DS), according to the recommendations of the American Society of Gastrointestinal Endoscopy (ASGE) of sedation administered by the anaesthetist for complex endoscopic procedures [8].

The widespread use of DS during ERCP is based on little data to support it compared to traditional general anaesthesia (GA), and to date, current standards of care have not adopted a general practise of GA or DS for ERCP [9]. In this regard, a meta-analysis reported that propofol in advanced endoscopic procedures is associated with shorter recovery times and better quality of sedation and amnesia level, without an increased risk of cardiopulmonary complications, with better sedative conditions achieved by propofol-opioid regimens than propofol alone during oesophageal procedures [10]. The rapid onset and short half-life of remifentanil, a powerful opioid, facilitate the titration of the drug dose according to the needs of each patient [11]. The combination of remifentanil and propofol for deep sedation reduces movements accidents, coughs, and hiccups during colonoscopy [12, 13]. However, some studies have also highlighted the negative impact of opioids (especially Remifentanil) on the gastrointestinal tract during advanced endoscopic procedures [14–20].

This study aimed to evaluate the feasibility and safety of continuous intravenous infusion of propofol and remifentanil for deep sedation of spontaneously breathing patients undergoing ERCP, regardless of the ASA risk classification of the patients.

## Methods

### Study design and population

This is a single-centre observational prospective cohort study conducted at ‘Tor Vergata’ University Hospital ‘Tor Vergata’ in Rome, Italy.

All consecutive patients undergoing ERCP from January 2021 to June 2021 were prospectively included (Table 1). Written informed consent was obtained from all patients who met the inclusion criteria.

**Table 1:** Population

PARAMETERS	VALUE
**PATIENTS**	106
**GENDER (male/female)**	54/52
**AGE, mean (SD)**	71 (12,37)
**BMI, mean (SD)**	25,81 (20,14)
Underweight, < 18,5 (%)	2,91
Normal, 18,5–24,9 (%)	35,89
Overweight, 25–29,9 (%)	38,8
Obesity I, 30–34,9 (%)	12, 61
Obesity II, 35–39,9 (%)	0
Obesity III, > 40 (%)	2,91
**SMOKE (%)**	
Yes	11,9
No	43,35
Ex	17
**ALCOHOL (%)**	0,92
**COMORBIDITIES**	
Ictus Cerebri (%)	6,84
High Blood Pressure (%)	63,19
Metabolic Disorders (%)	25,11
Chronic Kidney Disease (%)	12,18
Chronic Liver Disease (%)	12,04
Chronic Cardiac Disease (%)	42,75
Anticoagulant Therapy (%)	13,6
Antiplatelet Therapy (%)	33,12
Benzodiazepines (%)	2,58

The inclusion criteria were as follows:

- elective ERCP procedures,
- Patients with a normal state of consciousness and spontaneous breathing before the procedure,
- patients who were able to sign informed consent,
- procedures performed in the inpatient daily regime or ordinary inpatient,
- men and women with a score between I and III according to the American Society of Anesthesiologists (ASA) physical status classification system,
- All patients who did not meet the exclusion criteria.

The exclusion criteria were:

- men and women with a score IV or V according to the ASA physical status classification system;
- men and women with a previous anesthesiological evaluation in which a difficult intubation is expected (the evaluation was carried out using the Mallampati score and the physical examination that allowed identification of an interdental distance of less than 4 cm and a thyro-chin distance of less than 6.5 cm, rigid and wide neck, elusive chin).
- a known allergy to drugs to be used for sedation,
- emergency procedures,
- less than 18 years old,
- pregnancy,
- basal conditions that enhance tolerance to sedative and hypnotic drugs (such as drugs or alcohol history of abuse),
- lack of consent.

The ERCPs carried out under emergency conditions in our hospital mainly concern fragile and compromised patients (generally positive history of liver transplantation, pancreatic tumors, hyperbilirubinemia). In these cases we tend to move away from the line of conduct of the aforementioned protocol, rather we opt for a more personalized sedation by type of drugs and dosages, often assisted by the use of high flows through nasal cannulas (if the patient’s impairment mainly concerns a respiratory deficit) or we proceed directly with general anesthesia with orotracheal intubation, if the first approach should be judged unfeasible.

### Endpoints

Primary outcome: to assess the safety of the procedure by describing the occurrence of hemodynamic instability, arrhythmias, and gas exchange dysfunction (that is, hypoxemia monitored by the SpO_2_ and SpO_2_/FiO_2_ ratio, and/or hypercapnia monitored by EtCO_2_) throughout the procedure.

Secondary outcomes: to assess the feasibility of deep sedation in spontaneous breathing by describing the prevalence of signs of inadequate level of sedation, such as movement, coughing, and hiccups, and b) the time needed to achieve the target level of sedation and that for recovery from anaesthesia.

As a subgroup analysis, the difference in peripheral oxygen saturation was measured between the various classes of BMI, distinguishing overweight patients from normal or underweight patients.

To minimise bias due to the procedure, both endoscopic and anesthesiologic, the same operating team and anesthesiologist were used for all patients.

### Endoscopic procedure

ERCP was performed by an experienced operator for benign and malignant biliopancreatic diseases. Therapeutic procedures (e.g., sphincterotomy, stone extraction, biliary stent, etc.) were performed at the discretion of the endoscopist according to the clinical indication. Rectal non-steroidal anti-inflammatory drugs were administered to prevent post-ERCP pancreatitis, if not contraindicated. Adverse events were defined according to consensus criteria [8].

### Anaesthetic protocol

In the pre-procedure phase, two peripheral venous accesses were cannulated. According to the Perioperative Fluid Management in the Enhanced Recovery After Surgery (ERAS) protocol, the patient fasted from clear liquids for at least two hours before induction of anaesthesia and Ringer Lactate was used to maintain euvolemia.

During the endoscopic procedure, each patient was placed supine, with the chest tilted 30°.

All patients received midazolam, propofol, and remifentanil intravenously to induce and maintain DS. The drugs were administered in the following doses:

- INDUCTION: midazolam 0.06 mg/kg and propofol 0.5 mg/kg, both in single dose administration.
- MAINTENANCE: continuous infusion of propofol 1% at a rate of 4 mL/h and remifentanil 50 mcg/mL at a rate of 3.5 mL/h, both delivered by a Braun syringe pump. The drugs were titrated in each patient according to the ideal body weight (IBW).

The following parameters were monitored during DS: SpO_2_ (peripheral oxygen saturation), FiO_2_ (fraction of inspired oxygen), NIBP (noninvasive systemic blood pressure), HR (heart rate), number of breaths per minute, neurological functions with the use of the bispectral index (BIS^™^) to determine depth of anaesthesia, and EtCO_2_ (partially exhaustive CO_2_ end pressure) by a properly calibrated device (Smart CapnoLine® Guardian^™^ O_2_ Microstream®) to continuously assess the ventilatory status.

At the end of the procedure, SpO_2_, respiratory dynamics (such as respiratory excursion), the number of breaths per minute, EtCO_2_, and hemodynamic stability (NIBP, HR, any intraprocedural hypotension, rhythm variations) were assessed. The patient’s state of consciousness was assessed both before and after the procedure, using the Richmond Agitation-Sedation Scale (RASS) in addition to accurate recording of the time of drug administration, with respective dosage, onset of action and offset, and the VAS Scale (Visual Analogue Scale) was used to estimate the level of post-procedural pain, both tested when the patient could execute simple commands; If discordant values were found in the absence of sedation/hypnosis and agitation according to RASS or VAS values > 4, they were tested again after half an hour, according to the guidelines of both assessments. During the procedure, deep sedation was performed according to the routine methodology.

### Data collection

Data were collected on individual patient cards that were summarised in an anaesthetic record system such as a Microsoft Excel database; this included physical status, medical history, assessment of the risk score of the American Society of Anesthesiologists (ASA), home therapy, any adverse event recorded in previous anaesthesia, recent blood test results, recent ECG, and preoperative imaging (chest X-ray) where necessary. It also included the amount in milligrammes (mg) of drugs administered, induction time, discharge time, and vital parameters.

### Statistical analysis

The normal distribution of continuous variables was assessed using the Shapiro-Wilk test.

Continuous variables with normal distribution are presented as mean ± standard deviation (SD) and were compared using Student’s t test in two groups, analysis of variance and covariance (ANOVA), or Tuckey post-hoc test for mean pairwise comparisons in more than two groups as appropriate.

Continuous variables with non-normal distribution are presented as median and interquartile range (IQR) and were compared by the non-parametric Wilcoxon rank-sum test or Kruskall-Wallis rank test for multiple comparisons as appropriate.

Categorical variables were presented as the number of patients or percentages.

In light from the evidence of the study by *Smith et al.* [21]. in which the rate of occurrence of major complications was reported as 10%, considering 95% CI and given the possibility of a type I error, an adequate sample size was calculated as 83 patients.

P < 0.05 was considered statistically significant.

Statistical analysis was performed using Stata/BE 17.0 (StataCorp LLC).

## Results

During the study period, a total of 114 patients underwent ERCP: 106 (52 females/54 males; mean age: 71 ± 12,37 year.) were enrolled in the study, which met the inclusion criteria, while 8 patients were excluded.

The mean body mass index (BMI) of the patient group was 25.81, therefore, the majority were in the overweight range; in particular, 2.91% of the patients were underweight, 35.89% were normal weight, 38.8% were overweight, 12.61% were in the obesity class I range and 2.91% in that of obesity class III; 11.9% of the patients were smokers, 43.35% denied smoking habit and 17% were former smokers. Only 0.92% of the patients reported drinking alcohol.

Regarding medical history, 6.84% of the patients had a history of stroke, 63.19% had systemic arterial hypertension, 25.11% had type II diabetes mellitus, 12.18% had mild to moderate chronic renal failure, 12.04% had liver and / or biliary diseases, 42.75% had a history of ischemic heart disease. 13.6% were on anticoagulant therapy and 33.12% were on antiplatelet therapy. Only 2.58% took benzodiazepines at home.

The median PAS intraprocedural and postprocedural was 140 (125–150) mmHg and 139 (125–150) mmHg, respectively, p = 0.002 (Table 2).

**Table 2:** Cardiovascular and respiratory parameters

PARAMETERS	PREOPERATIVE median (IQR)	INTRAOPERATIVE median (IQR)	POSTOPERATIVE median (IQR)	*p-value*
**Heart Rate (bpm)**	75 (70–80)	75 (65–80)	75 (65–80)	0.24
**SpO**_**2**_ (%)	98 (96–99)	98 (97–99)	98 (97–99)	0.44
**SpO**_**2**_/**FiO**_**2**_	467 (452–471)	250 (245–278)	467 (457–476)	***0.0001***
**PAS (mmHg)**	148 (132–160)	140 (125–150)	138,032 (106)	***0.002***
**PAD (mmHg)**	80 (70–85)	72 (70–80)	71,280 (106)	***0.004***
**PAM (mmHg)**	100 (93–110)	94 (87–103)	104,656 (93)	***0.005***
**BIS**	-	61 (55-67.5)	-	*-*

written in bold: significant p-values

bpm: beats per minute

mmHg: millimiters of mercury

IQR: interquartile range

The median PAM intraprocedure and postprocedure was 94 (87–103) mmHg and 95 (88–101), respectively, p = 0.005 (Table 2).

The median HR intraprocedure and postprocedure was 75 (65–80) bpm and 75 (65–80) bpm, respectively, p = 0.24.

The median intra- and postoperative SpO_2_/FiO_2_ ratio weas 250 (235–285) and 467 (457–471), respectively, p = 0.0001.

During the procedures, the patients had a mean EtCO_2_ of 33.8 mmHg and a mean BIS of 60.

Regarding intraoperative drug use, 3.82 mg of midazolam were administered on average, with a total dose of 4.022, to ensure DS in induction; however, an additional dose was required in approximately 11 of 106 patients.

Regarding propofol induction, an average dose of 68.2 mg was infused, its onset, on average, was 63 s, thus defining the time required between induction and the start of the endoscopic procedure. To maintain deep sedation, propofol was administered through an infusion pump at an average rate of 5 mL/h and remifentanil with a mean dose of 0.038 mcg/kg/min. At the end of the procedure, flumazenil was administered averaging 0.3 mg and the patient’s recovery time was averaged 92 s. The mean estimated procedure time, from induction of anaesthesia to awakening of patients, was 69.35 min.

**Fig. 1:**
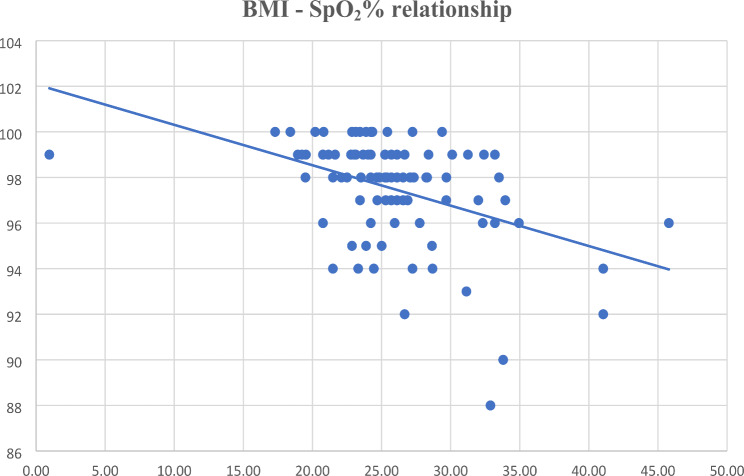
Intraoperative BMI-SpO_2_ relationship. In 88.5% of our patients with BMI > 30 the intraoperative SpO_2_ was found to be lower than that recorded in normal or underweight patients.

We found that in 88.5% of patients with BMI > 30, intraoperative SpO_2_ was lower than that recorded in normal or underweight patients (Fig. 1).

The overall duration of the procedure performed under deep sedation did not significantly affect hemodynamic stability (in 97% of the patients investigated in the study).

Analysing the different minor adverse reactions that have occurred (Table 3), we distinguish:

**Table 3:** Adverse Events

ADVERSE EVENT	%
Movements	25
Cough and hiccups	20
Hypotension	3
Desaturation	2
Arrhythmia	2
Secretions	1
Vomiting	1

- accidental movements: 25%.
- cough and hiccups: 20%.
- hypotension: 3%.
- desaturation: 2%.
- heart rhythm disorders: 2%.
- excess secretions: 1%.
- vomiting 1%.

In two of 106 patients (1,88%), the conversion from DS to GA had to be done for greater safety, given the condition of the patient. In these patients, after an irritant stimulus on the airways, probably deriving from endoscope manoeuvres (negative history of altered bronchial reactivity), an irritating cough arose, associated with bronchospasm, with concomitant difficulty in re-establishing SpO_2_% values comparable to those previous. Once the acute problem was solved with the appropriate manoeuvres and with the administration of beta2-agonists, it was decided to proceed with the operation by performing an orotracheal intubation under general anaesthesia. As a side note, we can point out that in both cases the ERCP procedures proved to be particularly arduous, probably due to the anatomical peculiarities of the subjects.

There were no serious complications such as major cardiac and respiratory events (cardiac arrest, respiratory arrest, etc.) (Table 2). As follow-up, we distinguish patients as hospital residents or from outside the hospital: Residents were redirected to the ward they belong to within 30 min of reaching a RASS value of 0, while patients from other facilities were left for observation in the recovery room, 60–90 min before being discharged as fully recovered.

## Discussion

ERCP is increasingly performed for therapeutic indications of varying complexity. The duration of the procedure and the complexity of the planned therapeutic intervention make effective sedation an integral part of achieving technical success. There has been an increasing trend toward endoscopic procedures performed with GA or DS provided by a specialist [22, 23]. Therefore, most endoscopic procedures, including those performed in patients in this study, are performed with anaesthesia assistance in those considered to be low to high risk (ASA class 1, 2 or 3) or at the patient’s request [24]. The American Society of Gastrointestinal Endoscopy (ASGE) suggests that anaesthetist-administrated sedation may be beneficial for ERCPs [8].

Our primary endpoint was to determine whether pre-selection for DS, regardless of the patient’s risk factors and the intended therapeutic intervention, had a good safety and efficiency profile, including resource management. The presence of an anaesthetist is necessary and mandatory during the performing of the procedures, regardless of the chosen management. In general, the AEs were not significantly burdensome and complex, such as discontinuing the procedure or permanently interrupting treatment. The low DS failures during ERCP and the reduced equivalent AE are probably due to the good anaesthetic plan of the drugs used, the reduced hemodynamic and respiratory impact on the patients, and the continuous monitoring of vital parameters. According to published evidence in this field, our study confirms the safety of deep sedation in spontaneously breathing patients using propofol infusion during ERCP, also allowing also quick recovery. As our results show (Table 2), the highest percentage of minor adverse events is due to awakening movements during the procedure (25%), probably due to a slow progressive drug titration to reach the target of the patients, particularly in more fragile patients (ASA 3), or events such as coughing or hiccups (20%), adverse effects described in the propofol data sheet as common (> 1/100, < 1/10) during induction, probably due to a higher manual infusion rate. The other adverse effects occurred cumulatively in 9% of the cases, which is a small percentage; we would like to emphasise that events that could be considered prodromes of major situations occurred in a very low percentage in our study: particularly arrhythmias in only 2% of the patients and vomiting in only 1%.

To best assess the secondary results of the study, we must first analyse the vital parameters of the patients throughout the endoscopic procedure. Hypercapnia is slowly established and pCO_2_ values less than 70 mmHg allow good procedural tolerability. A 100% increase over the baseline value results in a 50% reduction in alveolar ventilatory pressure, such as a reduced arterial PaO_2_, creating respiratory distress. From the data obtained from the examined patients, it can be seen that, in our study, EtCO_2_, an indirect measure of increased pCO_2_ in the blood, remained within safe ranges, with an average among patients of 33.833 mmHg; When breathing, it can be seen how the anesthesiologic plan of our deep sedation led to desaturation in a small percentage of patients (2%), also due to manoeuvres - such as aligning the upper airways for optimal air passage, and placing the patient in a correct sniffing position – that we did not include among the resolutions of adverse events, as these can be considered standard components to maintain optimal airway management, resulting in adequate oxygenation for all patients undergoing sedation [25].

The low rate of desaturation is also due to an optimal patient position on the endoscopic couch: It is crucial to position the patients in supine decubitus, with the chest tilted 30 degrees, so as to significantly reduce adverse events (vomiting, regurgitation, secretions, inhalations of water for washing the biliary tract by the operator) and maintain a residual functional capacity (CFR) close to 2 L, reducing respiratory distress. From a upright to a supine position, the CFR is reduced by 50%.

Blood pressure (whether systolic, diastolic, or mean is taken into account) during the interventional procedure also remained stable, with no major hypotension cones in the patients (3%) and without affecting heart rate, maintaining a steady pulse on average and within safe profiles; Speaking of hypotension, the use of low volume intravenous fluids or occasional injections of vasopressors during DS cases, particularly after induction, were not considered adverse events if they did not require procedural interruption and did not affect the patient’s outcome.

All this is perfectly in line with previous additional evidence showing that, compared to GA, deep sedation with propofol infusion in spontaneous breathing was associated with fewer hypotensive episodes but more hypoxemic events. In terms of risk of cardiac arrhythmias, a review of the published literature has shown that deep sedation is associated with a lower number of cardiac arrhythmias compared to GA [26–28]. In a randomised controlled trial, comparing deep sedation and GA for ERCP in 200 patients at risk of sedation-related adverse events, the incidence of adverse events was higher in the sedation group in which 10% rescue GA was necessary. However, there were no significant differences between the two groups in the quality and duration of the procedure and recovery after it [21].

In our cohort, which also included patients with BMI > 35, conversion to GA was only needed in 2 cases (less than 2%). In other words, the decision about whether to use deep sedation or GA in each case depends largely on the characteristics of the patient, the procedural demands of the specific case, and the existence of comorbid conditions.

A closer examination of the time required for anaesthesia management merits to the selective use of DS for ERCP rather than the universal use of GA. As can be seen in our results, the average induction time, after infusion and propofol onset, is very rapid (63 s), making it clear that the time from patient hypnosis onset to the start of the procedure is just as rapid; furthermore, the procedure itself has an average duration of 69.35 min, perfectly within the range of times described in the literature for this type of intervention (30–90 min). Ultimately, patient awakening, after intravenous infusion of the relevant amount of flumazenil, is also quite prompt: in our study, we found that the average awakening time was 92 s.

To assess the patient’s actual arousal, the Richmond Agitation-Sedation Scale (RASS) was calculated: is a 10-point scale that measures the patient’s level of agitation or sedation, consisting of four levels of anxiety or agitation (from +1 to +4), a level to indicate a state of calm and alertness (0), and five levels of sedation (from -1 to -5) which culminates in “not awakened” (-5).

Our observational study reports that 4% of ERCP patients with awakened DS had an RASS score of 1, while the remaining number of patients had an awakened RASS of 0. In the first group of patients, 1 g of iv paracetamol was administered and a subsequent reassessment according to the RASS scale was performed at 30 min. All patients returned to a RASS value of 0. As mentioned earlier, we repeat that hospital patients were redirected to the ward within 30 min of reaching a RASS value of 0, while hospital patients were left to monitor and recover fully up to 60–90 min before discharge.

## Limitations

In conducting our study, we also noted possible weaknesses, limitations, or biases that we could not fail to point out.

First, this is a study conducted in a single hospital centre, which, although it may gather a good pool of users and patients, is still a limited environment. Second, the sample examined could be considered small and without a control group, which also limited the possibility of conducting a randomised trial, allowing us only to conduct an observational study. Finally, data loss due to human error (failure to transcribe data, room staff changing from day to day or even between procedures) leads to greater difficulty in composing the statistics.

Another potential weakness of our study is the lack of a rigidly defined set of preprocedural criteria. The paucity and heterogeneity of data on the efficacy and safety profile of DS versus GA for patients undergoing ERCP establish the need for more precise guidelines regarding the extent to which anaesthesia services should be involved.

## Conclusions

In conclusion, considering the limitations mentioned above, our study found that deep sedation in spontaneous breathing during ERCP met good quality standards and was shown to be feasible and safe. It may offer comfortable sedation to patients in the absence of significant adverse events and it may reduce the time related to anaesthesia during procedures, avoiding endotracheal intubation.

However, more evidence from randomised controlled trials is needed to confirm these data.

## Data Availability

All data are present in the results and tables. Further enquiries about data can be obtained from the corresponding author on reasonable request.

## Abbreviations

AE: Adverse Event
ASA: American Society of Anaesthesiologists
ASGE: American Society for Gastrointestinal Endoscopy
BIS: Bispectral Index
BMI: Body Mass index
CFR: Residual Functional Capacity
DS: Deep Sedation
ECG: Electrocardiogram
ED: Digestive Endoscopy
ERAS: Enhanced Recovery After Surgery
ERCP: Endoscopic Retrograde Cholangiopancreatography
ESGE: European Society for Gastrointestinal Endoscopy
EtCO_2_: End-tidal Carbon Dioxide
FiO_2_: Fraction of Inspired O_2_
GA: General Anaesthesia
HR: Heart Rate
NIBP: Non-invasive Blood Pressure
PAD: Diastolic Arterial Blood Pressure (DBP)
PAM: Mean Arterial Blood Pressure (MBD)
PAS: Systolic Arterial Blood Pressure (SBP)
pCO_2_: Partial Pressure of Carbon Dioxide
RASS: Richmond Agitation-Sedation Scale
SD: Standard Deviation
SpO_2_: Peripheral Oxygen Saturation
VAS: Visual Analogue Scale
